# Review of mobile applications for optimizing the follow-up care of patients with diabetes

**DOI:** 10.1007/s42000-018-0062-0

**Published:** 2018-10-13

**Authors:** Nikolaos Th. Ersotelos, Andrew N. Margioris, Xu Zhang, Feng Dong

**Affiliations:** 1 School of Computer Science and Technology, University of Bedfordshire, University Square, Luton, Bedfordshire, LU1 3JU UK; 2 Department of Clinical Chemistry, Medical School, University of Crete, 71110 Heraklion, Crete Greece

**Keywords:** Diabetes, Insulin, Mobile applications

## Abstract

**Background:**

Several smartphone applications aim at facilitating communication between patients and healthcare providers. In this review, we evaluate and compare the most promising applications in the field of diabetes mellitus (DM) and obesity. Most applications monitor body weight, fasting or postprandial blood glucose, glycosylated hemoglobin (Hgb) A1c (HgbA1c), and units and types of insulin used.

**Methods:**

Nine clinically tested applications and two Web platforms were grouped into three categories that were evaluated and compared. Group 1 included seven applications focusing mainly on monitoring DM, fitness and weight, blood glucose levels, and HbA1c. Group 2 included two applications that focus on insulin dosage calculators and glucose self-monitoring tests. Group 3 included two web-platforms that interact with patients via SMS (short message service) messaging.

**Results:**

A common feature of the applications examined was the limited number of clinical parameters tested, the small number of subjects taking part in the evaluation, and the fact that the controls were not randomized. Furthermore, the interfaces of the applications varied and were not standardized. Finally, another common characteristic across applications was the lack of standardization of the interface and the overall structure due to language barriers, the devices usually having been designed around a specific language. Lastly, most applications lacked a critical mass of evaluators and were thus not worthy of being considered of serious clinical relevance.

**Conclusions:**

The current smartphone applications for DM are characterized by a limited number of participants, a small number of parameters, and a lack of standardization.

## Introduction

In 2015, in the UK, approximately 3.8 million people aged over 16 (about 9% of the adult population) were suffering from diabetes mellitus type 2 (T2DM) (https://www.gov.uk/government/news/38-million-people-in-england-now-have-diabetes, https://www.diabetes.org.uk/Preventing-Type-2-diabetes/Prevention/, http://www.diabetes.co.uk/diabetes-prevalence.html). Moreover, 11.9 million were at increased risk of developing insulin resistance and type 2 diabetes. It is also estimated that worldwide, around 220 million persons have T2DM, a figure that is expected to reach 366 million by 2030 [1, 2]. Obesity, sedentary lifestyles, and unhealthy eating habits represent the main causes of T2DM.

Approximately 95% of the daily management of T2DM depends on the patients themselves making multiple choices regarding the type of diet consumed, intensity of physical activity, levels of smoking and alcohol consumption, glucose monitoring type, and dosage of prescribed drugs, all this with minimal medical supervision [3, 4]. This requires extensive knowledge regarding the pathophysiology of T2DM, including the effects of the glycemic index (GI) of each food item, load of food intake (glycemic load), amount of exercise, the significance of hyper/hypoglycemias, dosage of antidiabetic medications, and amount and timing of insulin injections. It also requires problem-solving skills and behavioral changes to cope when things become unmanageable, which explains the increasing need for applications that permit closer interaction between the diabetic and healthcare-providers.

It should be noted that mobile applications now use GPS navigation, step trackers (accelerometer sensors), diaries, and digital cameras. A great number of patients with T2DM own smartphones in Europe, the USA, Asia, and the developing world, and very sophisticated phone devices are now widely affordable [3]. Statistical information about each individual patient, which is highly important for the management of the disease, is thus readily available to healthcare providers. There are number of such applications, including BG Monitor Diabetes, Diabetes in Check, Diabetes Pilot Pro, Diabetes Tracker, Diabetic Connect, Glucagon, Glucose Buddy, mySugr, and Diabetes Logbook, which have been designed to educate and encourage patients to adopt healthier lifestyles and to self-monitor their physical exercise, blood pressure, glucose levels, heart rate, body weight, and insulin dosages. Unfortunately, the validity of these applications has not been verified via extensive clinical tests and, consequently, limited confidence can be placed in their efficacy and safety.

## Methodology

To facilitate comparison, the applications have been divided into three major categories:

- *Seven lifestyle self-management applications for T2DM patients*: They mainly focus on monitoring daily caloric intake, type of food consumed, exercise, fasting blood glucose (FBG), postprandial glucose (PPG), and type of medications used. These health apps aim to motivate the users to adopt better lifestyle habits.
- *Two insulin dose calculators for T1DM patients*: We compare the accuracy and clinical suitability of two applications for calculating treatment, mainly focusing on insulin calculators for patients with T1DM. Because self-medication errors are recognized as a source of avoidable harm, these applications deserve particular scrutiny [5].
- Finally, *two text messaging intervention applications for T2DM patients*: Text messaging intervention is an approach designed to motivate mobile phone users to improve their health by receiving frequent reminders about exercise and blood tests and about improving the quality of their diet.

Each application was evaluated for (i) straightforwardness of installation and compatibility, (ii) clarity of language, (iii) simplicity of use, (iv) inclusion of daily health monitoring features, (v) educational value, (vi) sharing data with healthcare providers, and (vii) effectiveness of the intervention.

Each application was further examined from a statistical point of view providing to the reader means, standard deviation, and *P* values (e.g., mean ± SD or % improve/drop) wherever available. The clinical evaluation of each application includes patients’ fasting glucose levels, PPG levels, changes of hemoglobin HbA1c levels, and frequent hypoglycemic events.

## Applications for T2DM patients

All of the selected applications conform to the guidelines of the *Food and Drug Administration (FDA)* [6].

### bant2

**Fig. 1:**
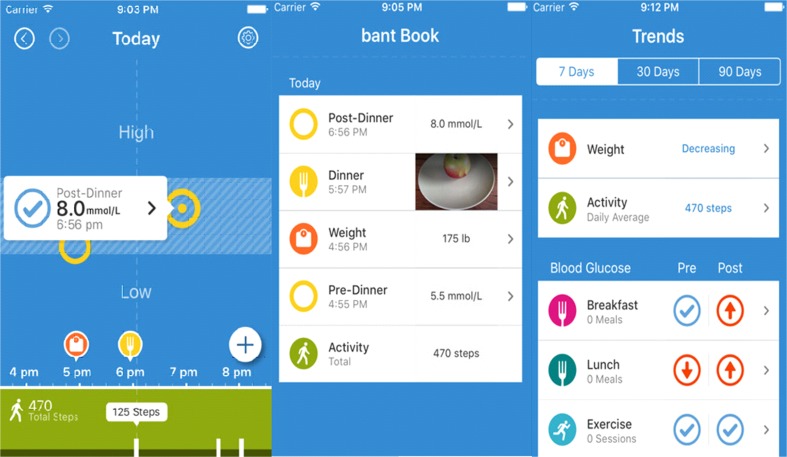
The “bant2” mobile application interface aiming in helping T2DM patients to monitor and maintain normal daily glucose levels. The green tab (bottom left) monitors steps. The middle tab shows the daily input statistic. The upper right-hand tab shows the patient’s current weight and steps activity. The lower right-hand tab shows the user’s daily blood glucose levels based on food intake and number of exercise sessions

The “bant2” application is easy to use, English is the interface language, and it is available for both Android and Apple devices. It was designed, via Bluetooth connection, to help monitor daily food intake, weight and blood glucose, and HbA1c. It can also be connected to several external devices and store the gathered data. We have found that the application exhibits several shortcomings including the following: (i) it cannot track caloric intake, which can only be deduced by pictures of the meals; (ii) the educational value of the interface is severely limited, and most users have commented negatively regarding it; (iii) its motivational value is limited; therefore, it may only be used as a tracking or reminding tool; and (iv) the stored data remain within the device; hence, the healthcare providers do not have access to the data since the device does not provide sharing facilities (Fig. 1).

#### Clinical evaluation of the application

A current clinical study is evaluating “bant2” application on 150 English-speaking T2DM participants with HbA1c > 7.5%, who do not take insulin. The principal analysis strategy was based on linear mixed models in the Statistical Analysis System (Cary, NC, USA) using the PROC MIXED procedure. The following parameters will be examined: (i) the effect of the application on glycemic control, (ii) effect of the application on lifestyle parameters, (iii) if it facilitates self-care activities, and (iv) if it improves several biochemical parameters of their diabetes [7, 8]. The clinical trials started in 2015 and are due to be completed by the end of 2018. Early results suggest that the application improved HbA1c levels, blood pressure, cholesterol, and total weight.

### Noom Health

The “Noom Health” mobile application is available only for English-speaking Android users. In contrast to “bant2,” “Noom Health” aims at educating users on various aspects of their disease. Through a combination of automated and human interventions, it teaches patients how to sustain their diet gains and activity levels via (i) a “virtual” coach that delivers timely, tailored, and motivational guidance; (ii) trained facilitators who lead “in-app” support groups to bolster patient engagement; (iii) a 500,000+-item international food database; and (iv) a color-coded calorie density feedback indicator regarding healthier food choices. It should be noted that a user wishing to access the application is asked to contact the “Noom Health” company and request a personalized ID. The installation process, according to users’ reviews, is rather cumbersome, as it necessitates a register-activation authentication process.

#### Clinical evaluation of the application

It was clinically tested in a pilot study on 62 overweight or obese insulin-resistant adults who received a 24-week “virtual diabetes prevention” program, with human coaching [9]. The main aim of this preliminary evaluation was to test the application’s weight loss efficacy. Samples were compared using the *t* test between non-starters and starters. It is of interest that multiple measurement analysis of variance (ANOVA) showed the effect of time on weight changes. Backward regression analysis examined whether engagement variables were able to predict weight loss at 16 and 24 weeks. In the clinical evaluation tests, eight participants were excluded from the first week for not following the instructions and, of the remaining 54, 36 completed the full treatment. Ad hoc ANOVA tests indicated that weight loss was significant at 16 weeks for starters (weight loss = − 5.40, SD = 4.43, *P* < 0.001) and completers (weight loss = − 6.00, SD = 4.34, *P* < 0.001). Weight loss was also significant at 24 weeks from baseline in starters (weight loss = − 6.22, SD = 5.00 *P* < 0.001) and completers (weight loss = − 7.01, SD = 4.83, *P* < 0.001). The main limitation of the clinical evaluation was that it lasted only 6 months and that the participants were not examined for persisting insulin resistance, glucose levels, or HbA1c.

### DialBetics

The “DialBetics” application is produced for Japanese-speaking mobile users as a pilot study by the Japanese Society for the Promotion of Science and was designed for T2DM patients to help them with their lifestyle self-management [10]. This application is similar to “bant2” as it can be connected via Bluetooth to several external devices for collecting several parameters, including daily blood glucose levels, blood pressure, body weight, and pedometer data; additionally, the data are not only stored in a calendar but also automatically transmitted to the Society’s doctors, who advise on intensive follow-up exercises, or, in cases of abnormal readings, additional or different treatment modalities.

#### Clinical evaluation of the application

The study of the clinical efficacy of the application should be considered preliminary, as a longer study will follow of more participants to validate the long-term effects. More specifically, this preliminary evaluation was composed of two groups in this trial aiming at determining safety and usability. Group 1 consisted of 54 participants with T2DM who were not on insulin injections, while ten participants with T2DM in Group 2 were on insulin treatment. After 3 months, the HbA1c of the first group using DialBetics was slightly improved, from 7.1 ± 1.0 to 6.7 ± 0.7%. The second group was followed up for only 1 month. The authors report a reduction of the insulin needed compared to baseline, but this was based on a limited number of participants, while the difference in HbA1c is borderline, from 7.0 ± 0.9 to 7.1 ± 1.1% (*P* = 0.019).

### Welltang

The “Welltang” mobile application [11] uses Chinese (Mandarin) as an interface language for both Apple and Android devices. It is characterized by three main features: (i) guidelines giving daily advice about diet, exercise, medication, how to self-monitor blood glucose levels; (ii) monitoring and measuring medication and assessing lifestyle activity effectiveness; (iii) daily recordings of diet, physical activities, glucose levels, and medicine dosages transferred to users’ accounts that are retrievable in the event of mobile failure; and,(iv) similarly to “DialBetics,” a professional version for doctors to monitor progress offers advice and information and adjusts prescriptions and dosages.

#### Clinical evaluation of the application

The clinical evaluation of the “Welltang” application is considered satisfactory, the sample consisting of 100 evenly randomized patients with T2DM between the ages of 18 and 74 recruited for a 3-month period to test the effectiveness of the application regarding changes of FBG levels, 2-h PPG, body weight, blood pressure, low-density lipoprotein (LDL) cholesterol, and frequency of hypoglycemic events. Statistical analysis was carried out with the SPSS package (SPSS Inc., Chicago, IL, USA). Prior to analysis, the distribution variables were tested to ensure that assumptions of normality were valid. An independent *t* test was used to compare the difference between two study groups, while a paired sample *t* test was used to compare changes from baseline to 3-month follow-up. The authors have found a decrease of HbA1c by 1.95% in the intervention group and 0.79% in the control group (*P* < 0.001). Eighty-four percent of patients in the intervention group declared their satisfaction with the application vis-à-vis the user-friendliness aspect.

### Diabetes Pal

**Fig. 2:**
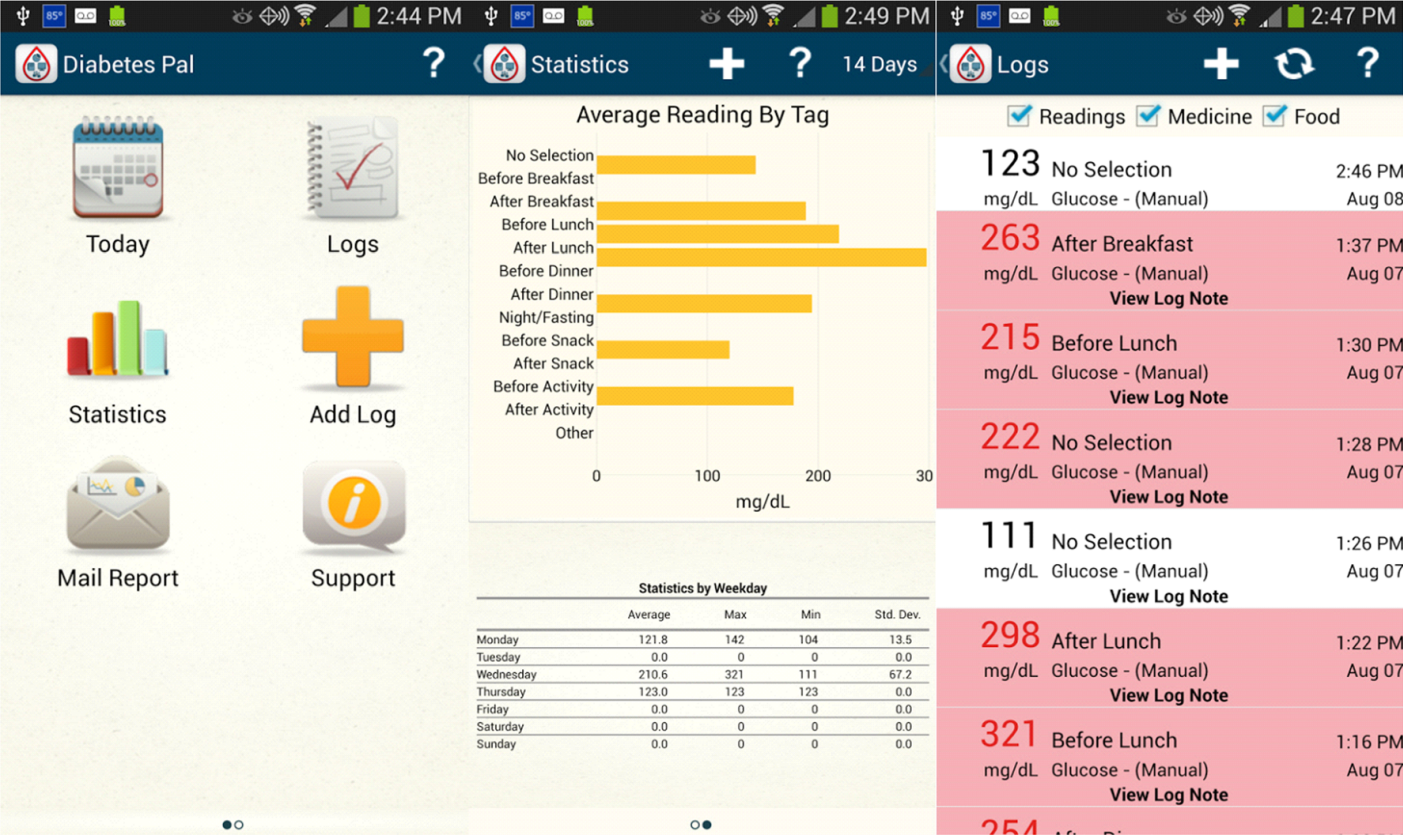
The interface of the mobile application Diabetes Pal. The top left tab is the app navigator. On the second tab, the patient can see his/her health status and activities for a period of 14 days. The panel along the right-hand side shows fasting and postprandial blood glucose levels

The advantage of the “Diabetes Pal” (Fig. 2) application is that it allows T2DM users to transfer glucose monitoring data either automatically via Bluetooth—such as by the Telcare BMG device—or manually to data-storing devices, including mobile devices and/or Web servers, allowing them to be accessed by healthcare providers. Patients can also select a specific time period from their diaries. Users’ reviews suggest that it is an easy application to use, while it can store glucose results and a wide range of medical and prescription information. Health providers can monitor their patients’ health status only if patients are willing to share their health information. It is available for both Android and Apple devices. The application also helps with hypoglycemia episodes by guiding the patient via comprehensive text instructions. The drawback of this application is that it can only track FBG levels but does not store other data, including lifestyle and physical activity, number of steps, caloric intake data, body weight, or hypoglycemic events.

#### Clinical evaluation of the application

A 24-week, randomized, open-label, parallel-group trial was conducted at the Singapore General Hospital between March 2013 and March 2015. The study was approved by the ethics board of the Singapore Health Services.

The first group consisted of 33 insulin-naive T2DM patients. However, three handicaps can be seen, namely, the number of participants was small, there was no control group, and the number biochemical parameters evaluated was small. All the participants received a one-time individual educational session with a diabetes nurse educator. The participants were instructed to start with 10 units of insulin detemir at bedtime, self-monitor FBG daily, and self-titrate insulin every 3 days using a prescribed algorithm to reach a target FBG of 72–126 mg/dL (4.0–7.0 mmol/L). The algorithm was based on the mean FBG (mFBG) over 3 days (10 units of insulin); when mFBG exceeded 180 mg/dL, the dose was also exceeded by 4 units and when mFBG was 126–180 mg/dL was exceeded by 2 more units. If FBG was < 72 mg/dL, the dose was reduced by 4 units. Up-titration of insulin dose was continued until the patient reached the target FBG or the predetermined maximum insulin dose (0.6 units/kg) allowed for self-titration (i.e., algorithm end point), whichever occurred first. Patients in the intervention group (*n* = 33) entered FBG readings into the app daily; the app responded with a suggested insulin dose. Each patient’s maximum dose was preset and locked in the app as a safety measure. It must be mentioned as limitations that the patients were unable to edit typographical errors in their FBG inputs, a field for actual injected dose was not available, and the application was not usable offline.

The second group consisted of 66 patients. Twenty-two of them were women; age was 53.3 ± 7.4 years; duration of diabetes was 12 ± 8 years; the HbA1c was 9.9 ± 1.8%. The authors found an average drop of HbA1c levels before and after 6 months from 9.9 to 8.8%, with no statistically significant difference between the groups (*P* = 0.88) [12]. While most participants had experienced at least one confirmed hypoglycemic event in the past, no new episodes were recorded during the trial period.

### PilAm Go4Health

The application “PilAm Go4Health” [13] started as an attempt to evaluate the use of smartphone external devices in the management of patients with T2DM and, more specifically, how to help them lose weight. This application is similar to the “bant2 and DialBetics” applications. It can be connected to Bluetooth devices, such as Fitbit, which tracks step activities. It does not store the type of food consumed, total caloric intake, and biochemical parameters.

#### Clinical evaluation of the application

This study was a 3-month-based pilot randomized controlled trial (RCT) with an active waitlist control and a 3-month maintenance design. The objective of this trial was to assess the feasibility and potential efficacy of the PilAm Go4Health intervention to reduce risks for metabolic syndrome in Filipinos with T2DM. Inclusion and exclusion criteria were based on the American Heart Association metabolic syndrome risks, diagnosis, and management, as well as the DPP trial. Forty-five T2DM Filipino-American patients took part. Participants were randomized to an intervention group (*n* = 22) or active control group (*n* = 23), and analyses of the results are underway. The participants could track and store their daily steps and upload diet recommendations, recipes, and activity results via a Facebook group page. The application does not provide educational information about their condition. Once every 3 months, each participant has to visit the hospital for training, coaching support, and progress evaluation. To evaluate the effectiveness of this approach, blood readings were taken in the third and sixth months, with results showing an average fall of HbA1c levels from 7.4 to 6.9%. At the start, the average of body mass index (BMI) was 30.2 + 4.9 and after 6 months decreased to 28.4 ± 3.3. Comparisons of baseline characteristics demonstrated two statistically significant differences (*Pm* < 0.05) between the intervention and control groups.

### ONE DROP

The “ONE DROP” is a coaching and educational application for Apple users, recognized by the American Diabetes Association. Twelve lessons are provided over a 9-week period. These lessons are aimed at providing instructions and recommendations to help the users monitor their health status daily. It provides the same features as the already mentioned applications, plus a communication feature via SMS with live coaching by a certified diabetes educator (CDE) member for 24 h a day/5 days a week. The coach is available for the first 9 weeks’ educational period to answer users’ questions. Additionally, the user can share his/her personal health records and achievements with other users in order to motivate or to be motivated.

#### Clinical evaluation of the application

One hundred forty-six patients tested the ONE DROP application for 12 weeks [14]. Seventy-one percent were female and 25% were black or Hispanic, diagnosed with T2DM. On average, participants actively used the app on 55 days over the 84-day (12-week) study period. An active day was defined as a day when the participant interacted with the application. Of the participants who completed the final assessment, 94% reported that the diabetes mobile application with in-app coaching helped them to manage their diabetes satisfactorily. Overall, study participants sent an average of 14 messages to their coaches. Approximately 78% of the participants who completed the final assessment found the educational content and lessons helpful, and 73% reported that they thought their coach was motivating and provided useful strategies. The two-tailed paired *t* test and a nonparametric signed-rank test compared mean and median change in HbA1c from baseline to study end. The authors reported an improvement of HbA1c by − 0.86% (*P* = 0.0003) among study completers (*n* = 127), − 0.96% (*P* < .001) among active users of the app and coaching program (*n* = 93), and − 1.32% among active users with a baseline HbA1c ≥ 9.0% (*P* < 0.001) (*n* = 53).

## Applications for T1DM patients (insulin dose calculators)

There are few applications that aim at helping patients with T1DM to calculate their daily insulin needs. Accurate calculations are based on factors such as (i) current and target blood glucose, (ii) carbohydrate-to-insulin ratios, (iii) total grams of carbohydrate in meals, and (iv) insulin already in the system [15]. However, it is difficult for patients to take all these factors into account when calculating dosages. Currently, numerous applications for Android and Apple devices claim to have such calculations; however, the majority have not been tested by clinical trials; thus, patients may risk overdosing or suffering hypoglycemic events. The following section presents clinically tested smartphone insulin dose calculator applications.

### RapidCalc

**Fig. 3:**
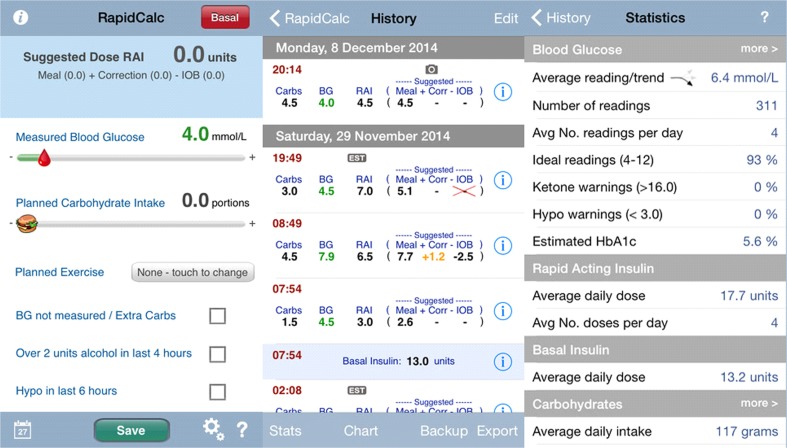
RapidCalc mobile application interface. The top left tab gives input information. A rough daily estimate of consumed food/cal and blood glucose metrics, plus other important information enabling a calculation of the insulin dose required. A disadvantage is that it has no calories tracker. The middle column shows insulin dose calculations at different times; the right-hand column gives statistics for a specific period

The application “RapidCalc” (Fig. 3) was designed for English-speaking Apple device users only. It provides a full 90-day record of insulin doses, carbohydrate intake, and glucose blood readings that can also be exported via email for archiving or further analysis. Charts and summary statistics help with blood glucose control, identifying trends, and even estimating HbA1c. Additionally, by entering blood glucose and food carbohydrate values, it can suggest meals and corrected bolus insulin doses. Although it is easy to use, it has several limitations: it does not (i) provide a database of food caloric intake, (ii) collect activities tracker data, or (iii) educate or motivate users.

#### Clinical evaluation of the application

The clinical evaluation of this application was elementary. Only seven patients with T1DM took part and for only 4 weeks. The interview group had previously received a flexible multiple daily injection (MDI managing complex mathematical calculations to determine insulin doses) education program by attending the Dose Adjustment for Normal Eating (DAFNE) program. The DAFNE program aims to equip graduates with evidence-based insulin self-management skills based on carbohydrate counting and insulin algorithms and includes insulin adjustment strategies for physical activity, illness, and when consuming alcohol.

It has been reported by the authors that the participants experienced a rapid improvement of their insulin needs compared to a control group using manual methods [16]. However, the authors provide no statistical evaluation. All the users trusted the application’s calculations of insulin doses, although its 90-day storage feature provided doctors with limited data regarding the patients.

### IDM

The University of Alberta’s “IDM” for Android and Apple devices is another T1DM application with features similar to those of “RapidCalc,” plus a Web platform supporting viewing of diary statistics.

#### Clinical evaluation of the application

It was tested on 31 patients, of whom only 18 completed the 4-week test period [17]. Results are expressed as mean ± SD or median and 25th and 75th interquartile range (IQR). Student’s *t* test was used to assess differences between groups and a signed rank test when the normality test failed. Proportions were assessed using the *z* test, and all analyses were performed using Sigma-Stat (Systat Software, San Jose, CA, USA). The dropout rate suggests a major weakness, namely, a possibly confusing layout or users’ difficulty in understanding the insulin calculator. The median HbA1c levels fell in a borderline manner from 8.1 (range 7.5 to 9.0) to 7.8% (6.9 to 8.3; *P* < 0.001) for those completing the test.

## Text messaging intervention (SMS)

### SMS to improve glycemic control among patients

This approach was designed to motivate mobile phone users to improve their health by receiving reminders about exercise and blood tests and ways to plan better diets. The limitation of this approach is that patients cannot monitor their health status, and there is no interactive communication with the healthcare providers. This approach was first introduced by the Detroit Partnership Diabetes Lifestyle Intervention and Health Sciences in Dhaka (Bangladesh) [18] and, while proving simple to use, left a lot to be desired.

#### Clinical evaluation of the approach

A total of 236 adult patients with T2DM (diagnosed within the previous 5 years) on oral medication therapy with access to SMS and attending the outpatient clinic of the Bangladesh Institute of Health Sciences in Dhaka, Bangladesh, were recruited (September 2013–August 2014) and randomly assigned to SMS intervention versus to standard care groups. Data were collected through face-to-face interviews with a structured questionnaire, anthropometric measurements, and blood tests for HbA1c using standard procedures. A total of 36 participants with missing HbA1c data were excluded from the analysis. All participants in the intervention group received 90 SMSs, based on the principles of behavioral learning theory, once a day, over a 6-month period. The average age of the participants was 48 + 9.7 years, and 54.2% were female. The average HbA1c levels at baseline was 8.4 + 2.6%. The mean difference of HbA1c from baseline to after 6 months (primary end point) was − 0.85 in the SMS group and − 0.18 in the control group. The difference between means was − 0.66 (*P* < 0.0001). Post hoc subgroup analyses suggested that the SMS intervention worked better in women and in those with a baseline HbA1c 0.8% and those with a shorter duration of diabetes.

### REACH

The application “REACH” (Rapid Education/Encouragement and Communications for Health) belongs to this category.

#### Clinical evaluation of the application

Thirty-six participants were recruited for the clinical evaluation with rather impressive results [19]. The participants’ average age was 52.4, 56% were female, and 63% were a racial or ethnic minority. About half were taking insulin. The starting HbA1c levels were approximately 8.2%, which was suppressed to 7.5% by the end of the trial period, while their weight loss averaged 5%. Unfortunately, the authors provide no statistical evaluation. It should be noted that similarly to the previous approach, frequent face-to-face meetings with the health providers were essential in order to obtain these results.

## Discussion

The bant2 application appears to be the easiest to use. The interface is in English, and it is available for both Android and Apple devices. It mainly aims at helping patients to monitor their daily food intake, weight gain or loss, fasting blood glucose, and HbA1c. We have found that the application has several shortcomings, including lack of a calories tracker, while the data it can store remain within the device; hence, they are not accessible to the healthcare providers. Unfortunately, the statistical evaluation of the application is still missing, since the evaluation is still in progress.

The Noom Health application is available only for English-speaking Android mobile users. It aims at educating the users on various aspects of their condition. The power of this application is that it provides a virtual coach (avatar) which delivers timely, tailored, and motivational guidance. In addition, this application offers a 500,000+-item international food database and a color-coded food calorie density list. A major shortcoming of this application is the preliminary evaluation of the application, which was tested only for 24 weeks on 62 overweight patients. The strength of this application is that it was tested thoroughly by *t* test and ANOVA.

The DialBetics is a Japanese mobile application which is similar to bant2. It stores data in a calendar and transmits them automatically to the healthcare providers. The clinical efficacy of this application is still elementary and needs to be improved. Based on the application’s clinical statistics, the difference achieved in HbA1c appears to be borderline.

The Welltang is a Chinese Mandarin application for both Apple and Android devices. It offers guidelines on a daily basis regarding diet, exercise, medication, and blood glucose levels. The performance of the Welltang is considered satisfactory. From the statistical point of view, the authors used the paired t test based on a 3-month follow-up. HbA1c decreased by 1.95% in the intervention group versus 0.79% in the control group (*P* < 0.001).

The strength of the Diabetes Pal application is that it enables users to send their glucose monitoring data directly to the healthcare providers via several methods. The application also helps with hypoglycemia episodes by guiding the patients. The main drawback of this application is that it can only store FBG levels. Moreover, although the clinical evaluation was conducted at the Singapore General Hospital, the number of participants was small, there was no control group, and the number of the chemical evaluated parameters limited.

The application PilAm Go4Health aims at helping patients with T2DM to lose weight. It is similar to bant2 and DialBetics. However, it does not store caloric intake and biochemical parameters. The statistical evaluation of HbA1c and BMI after 6 months showed the levels to be statistically significantly decreased, with a *P* < 0.05.

The “ONE DROP” was developed for Apple devices and is similar to the bant2, Welltang, and Diabetes Pal applications. The additional feature which it provides is the live coaching service by a CDE member for 24 h a day/5 days a week. Moreover, the user can share his/her personal health records and achievements with other users in order to motivate or to be motivated. The clinical evaluation was satisfactory for both the study completers (*P* = 0.0003) and the active users (*P* < 0.001).

The application “RapidCalc” was designed for English-speaking Apple T1DM device users only. Mainly, this application is an insulin dose calculator. It provides a statistical analysis for a full 90-day record of insulin doses, statistical analysis of carbohydrate intake, and glucose blood readings, which can also be exported via email for archiving or further analysis. On the other hand, major limitations were also found, as it does not provide a database of food caloric intake and it cannot collect activities and educate or motivate the users. Unfortunately, the authors did not provide efficient statistical evaluation.

The IDM application, with similarities to the RapidCalc, mainly also targets T1DM patients. Unfortunately, it was evaluated in only 31 participants. The authors have used Student’s *t* tests to assess the differences between groups. The high dropout rate also suggests a major weakness, namely, a confusing layout. The HbA1c levels dropped in a borderline manner.

While some applications (Table 1) attempt to only educate patients regarding diet, the Chinese “Weltang” also covers medicinal requirements, a necessary feature for those patients taking a multitude of medicines. Only three applications (“DialBetics,” “Weltang,” “Diabetes Pal”) provide the personal health providers with feedback and not in a real time mode and only after a certain period of time, during which the clinical needs of each patient may have changed drastically. Only the DROP One application provides real-time coaching by a certified CDE. It is also noticeable that neither the “Noom Health” nor the “PilAm Go4Health” applications track blood glucose measurements, which are essential features for both T1DM and T2DM patients.

**Table 1:** Provided features of each application

Application name	bant2	Noom Health	Dial Betics	Welltang	Diabetes Pal	PilAm Go4Health	One Drop	RapidCalc	IDM	REACH	SMS every day
Language	English	English	Japanese	Chinese	English	English	English	English	English	English	Bangladesh
Compatible (Apple + Android)	Both	Android	Android	Both	Both	Both	Both	Both	Both	Both (SMS)	Both (SMS)
T2DM or T1DM	T2DM	T2DM	T2DM (insulin)	T2DM	T2DM	T2DM	T2DM	T1DM	T1DM	T2DM	T2DM
Pedometer	Via Bluetooth	✓	Via Bluetooth	Via Bluetooth	X	Via Bluetooth	✓	X	X	X	X
Calorie counter	X	✓	X	X	X	X (manually upload)	✓	Manual import	Manual import	X	X
Body weight	Manual import	Manual import	Manual import	Manual import	X	X (manually upload)	Manual import	X	X	X	X
Insulin dose calc	X	X	X	X	X	X	X	✓	✓	X	X
Medicine dose	X	X	X	✓	X	X	X	X	X	X	X
Glucose measures	Via Bluetooth	X	Via Bluetooth	Via Bluetooth	Via Bluetooth	X	✓	Manual import	Manual import	X	X
Blood Pressure	X	X	Manual import	X	X	X	X	X	X		
Education	X	✓	✓	✓	✓	Diet advices	✓	X	X	X	X
Motivation	X	Virtual Coach	✓	✓	✓	✓	CDE	X	X	✓	✓
Doctors’ feedback	X	X	✓	✓	✓	X	CDE	X	X	X	X
Web platform	X	X	✓	✓	✓	Facebook	✓	X	✓	X	X

## Conclusions

All mobile applications presented in this review were designed to assist patients at all stages of diabetes. They mainly focus on monitoring daily caloric intake, type of food consumed, exercise, FBG, PPG, and medications intake. On the other hand, applications targeting insulin administration in either T1DM or T2DM patients concentrate mainly on the calculations of daily insulin units. Clearly, an application providing both facilities would be more versatile and clinically helpful. It is our impression that the sophistication level of the existing mobile applications for the follow-up of diabetic patients is still insufficiently streamlined, and there is a limited level of interaction between patients and health providers. The clinical evaluation of all these applications has been based on a limited number of patients, lack of parallel controls, for short periods of time, and assessing few parameters.

All participants in the clinical trial experienced a lowering of HbA1c levels by a significant degree. However, several negative factors need to be considered, including language barriers, a variety of database parameters, such as calorie trackers, and non-inclusion of a real-time platform enabling doctors to monitor their patients’ health status. Clearly, there is some way to go until the appearance of the ideal application that has all the necessary features installed.
